# Knowledge, Attitude, and Practices of Clinicians Towards Involvement of Physiotherapists in the Management of Obstetrics and Gynaecological Conditions: A Cross‐Sectional Study

**DOI:** 10.1002/hsr2.70851

**Published:** 2025-05-26

**Authors:** Seth Amponsah‐Tabi, Daniel Awli Mawunyo, Timothy Kwabena Adjei, Cosmos Yarfi, Amponsah Peprah, Maxwell Kankam, Charles Senaya, Jude John Annan, Roderick Larsen Reindorff

**Affiliations:** ^1^ Department of Obstetrics and Gynaecology Komfo Anokye Teaching Hospital Kumasi Ghana; ^2^ Directorate of Obstetrics and Gynaecology, School of Medical Sciences Kwame Nkrumah University of Science and Technology Ghana; ^3^ Department of Physiotherapy and Sports Science Kwame Nkrumah University of Science and Technology Ghana; ^4^ Department of Physiotherapy and Rehabilitation Sciences, School of Allied Health Sciences University of Health and Allied Sciences Ghana

**Keywords:** attitude, gynaecologists, knowledge, obstetricians, physiotherapists, practice

## Abstract

**Background and Aims:**

Delivery of holistic healthcare service is pivotal to achieving the World Health Organization's definition of health, hence the need to include the services of physiotherapists in the management of obstetrics and gynaecological conditions. The utilization of services of physiotherapists by attending clinicians depends largely on their attitude and knowledge of the crucial role physiotherapy plays in managing these conditions. The knowledge and attitudes of these clinicians on the involvement of physiotherapy in patient care in Ghana are unknown. The study aimed to determine the knowledge, attitudes, and practices of clinicians in Ghana towards the role of physiotherapy in managing these conditions.

**Methods:**

This was a cross‐sectional study undertaken among clinicians at the Obstetrics and Gynaecology (OBGY) Directorate of the Komfo Anokye Teaching Hospital. A total of 63 participants from the department were recruited for the study using convenience sampling within a period of 2 weeks. An adapted questionnaire from previous studies was used to collect data for the study. The data were entered into SPSS version 20 and analyzed using descriptive statistics (frequency, mean).

**Results:**

The 63 study participants included 15 (23.8%) consultant obstetrician gynaecologists, 9 (14.3%) senior specialists in OBGY, 18 (28.6%) specialists, 15 (23.8%) residents in OBGY training, and 6 (9.5%) medical officers in the OBGY directorate. They all had a general knowledge of the role of physiotherapists in obstetrics and gynaecological practice. However, they had low knowledge of specific conditions that can be treated by physiotherapists. Participants also failed to adequately utilize the services of physiotherapists in their practice.

**Conclusion:**

Clinicians in Obstetrics and Gynaecology Directorate at Komfo Anokye Teaching Hospital have general knowledge of the role of physiotherapists in obstetrics and gynaecological practice, but limited knowledge of specific conditions amenable to treatment by physiotherapists. Consequently, the services of physiotherapists in their practice were not adequately utilized.

## 1 Introduction

Obstetric and Gynecologic Physiotherapy is a subspecialty in Physiotherapy concerned with the promotion of gynaecological well‐being of the woman and also the well‐being of the mother throughout the child‐bearing period. This branch of medicine helps the woman to adjust advantageously to reproductive health issues as well as physical and psychological changes of pregnancy and the post‐natal period, so that the stresses of child‐bearing are minimized, thus ensuring optimal healthcare [1]. Research has shown a rise in obstetrics and gynaecological conditions worldwide [2] which include pelvic organ prolapse, dyspareunia, sexual dysfunction, urinary tract infection, uterine prolapse, obstetric fistula, obstetrics palsy, urinary incontinence, and fecal incontinence [3].

These conditions affect the physical well‐being of women as well as their psychological, financial, emotional, and social well‐being [4]. Such women are likely to adapt to sedentary lifestyles due to pain, and fear of aggravating symptoms, making them less productive [5]. This comes with associated economic implications on families and the nation [6]. These conditions, however, can be prevented, managed, and rehabilitated through physiotherapy [7, 8].

Delivery of an optimal healthcare service is pivotal to achieving a complete health status, hence the need to include the services of physiotherapists in gynaecology and obstetrics [7]. The utilization of such services by obstetricians and gynaecologists depends greatly upon their knowledge and attitudes on the role of physiotherapy in the management of such conditions [9]. It has been observed that gynaecologists and obstetricians who have adequate knowledge of the role of physiotherapy in the management of such conditions will not hesitate to refer patients for therapeutic interventions which will go a long way to facilitate recovery and promote the general well‐being of patients [10].

Research works undertaken in various countries found varying levels of knowledge and, attitude, and the practice of physiotherapist involvement in obstetric and gynaecological conditions. In Pakistan, 2 out of 3 gynaecologists were aware of the importance of physiotherapy pre‐childbirth, and post childbirth [7], while 9 out of every 10 obstetricians and gynaecologists in South‐western Nigeria referred patients to physiotherapists (Odunaiya et al. [9]).

Currently, the evidence on knowledge, attitude, and practices of gynaecologist and obstetricians towards the involvement of physiotherapists in patients' management in Ghana is unknown, giving rise to a literature gap. Efforts to ensure the holistic provision of optimum care to such patients will, however, be grounded on the awareness and prevailing knowledge of the attending clinicians in a tertiary facility setting, which will consequently influence their practices. Komfo Anokye Teaching Hospital (KATH) is a teaching hospital and therefore remains a significant avenue to both provide quality care and training of health professionals in healthcare delivery.

This study sought to determine the knowledge, attitudes, and practices of clinicians at Komfo Anokye Teaching Hospital toward the involvement of physiotherapists in the management of obstetrics and gynaecological conditions.

## 2 Methodology

### 2.1 Study Design and Setting

A cross‐sectional study was conducted among clinicians working in the Obstetrics and Gynaecology Directorate of the Komfo Anokye Teaching Hospital (KATH). KATH is the second largest teaching hospital in Ghana that receives referrals from over 8 out of the 16 regions in the country. The hospital is affiliated with the School of Medicine and Dentistry of Kwame Nkrumah University of Science and Technology and is also accredited for postgraduate training by the West African College of Physicians and Surgeons and the Ghana College of Physicians and Surgeons. It has over 1200 bed capacity and 4000 workers with varied professional backgrounds. KATH is located at Kumasi, in the Ashanti region of Ghana. The Obstetrics and Gynaecology Directorate of the hospital has a bed capacity of 160 and conducts about 9000 deliveries annually. The department is clinically manned by house officers, medical officers, residents in training, junior and senior specialist obstetricians/gynaecologists, and consultants as well as pharmacists and midwives of varying expertise and experiences.

### 2.2 Sample Size and Sampling

Using the Taro Yamane formula [n = N/(1 + N(e)2], aiming at a study power of 80%, a sample size of 67 was attained for the study. There was a population of 81 clinicians (house officers excluded) in the department at the time of the study. In all, 63 out of the 81 clinicians were recruited into the study using convenience sampling. Inclusion criteria to participate in this study comprised clinicians with at least 1 year of working experience at the OBGY department of KATH. Recruitment into the study was based on the availability and willingness of Obstetricians and Gynaecologists to give consent and participate in the study. Clinicians who were not at the post were excluded from the study.

### 2.3 Data Collection, Procedure, and Analysis

Data was captured from study participants using a structured questionnaire with open‐ended and closed questions under specific categorical sub‐headings ranging from socio‐demographic profile to knowledge, attitudes, and practices of clinicians on the involvement of physiotherapists in patient management. Knowledge of participants was self‐reported as poor, fair, good, very good, and excellent. Knowledge of conditions amenable to treatment by physiotherapists was assessed using a Likert scale, which is classified as strongly agree, agree, neutral, disagree, and strongly disagree. The attitude of participants was similarly assessed using a 1–5 Likert scale where 1 is poor, 2 is fair, 3 is good, 4 is very good, and 5 is excellent.

Clinicians were approached and contacted for inclusion, during the departments' usual clinical meetings. Those who consented were recruited into the study and made to fill out a self‐administered questionnaire which lasted for a maximum of 15 min. Data collection started on August 9, 2022 and ended on September 2, 2022. In all a total of 73 questionnaires were distributed and 63 were successfully retrieved. Data was coded, inputted into Microsoft Excel, cleaned, and exported to SPSS Statistics 20 for analysis. Descriptive statistics was done for categorical variables using frequencies and percentages, and for numerical variables using mean and standard deviations.

### 2.4 Ethical Considerations

The study received ethical approval from the Committee on Human Research, Publication and Ethics, School of Medical Sciences (SMS), KNUST, and Institutional Review Board of Komfo Anokye Teaching Hospital with registration numbers CHRPE/AP/271/22 and KATH IRB/AP/077/22, respectively. Each study participant filled a Participant Leaflet form after consenting to participate in the study. Each participant, therefore consented individually to participate in the study. Confidentiality and anonymity were ensured during data collection and processing.

## 3 Results

Out of the 63 study participants, a significant 82.5% were males. The median age of respondents was 38 years (IQR‐6). Our findings revealed that nine out of every ten participants were married. A cumulative 90.5% of the participants were either in training or practicing obstetricians/gynaecologists as illustrated in Table 1.

**Table 1:** Demographic characteristics of study participants.

Variable	Frequency (*N*)	Percentage (%)
Gender		
Male	52	82.5
Female	11	17.5
Age category		
30–35	18	28.6
36–40	28	44.4
41– 45	11	17.5
46–50	4	6.3
51–55	2	3.2
Religion		
Christian	60	95.2
Muslim	3	4.8
Marital status		
Single	6	9.5
Married	57	90/5
Education		
MBChB	16	25.4
Membership	28	44.4
Fellowship	19	30.2
Position		
Consultant	15	23.8
Senior specialist	9	14.3
Specialist	18	28.6
Resident	15	23.8
Medical officer	6	9.5

**Figure 1:**
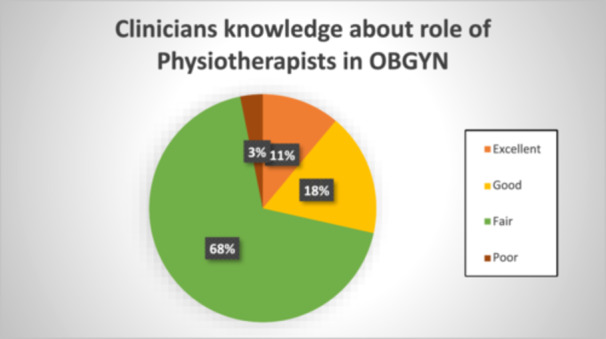
Clinicians' knowledge about the essence of the role of physiotherapist in obstetric/gynaecological practice.

In all, 11% of the clinicians rated their knowledge about the essence of the role of physiotherapy in obstetrics practice as excellent, while 68% rated theirs as fair as illustrated in Figure 1.

Over 95% of clinicians indicated they agree physiotherapy is needed in conditions of muscle weakness, 76% for sexual dysfunction, 65% for uterine prolapse, and 54% for urinary incontinence (Table 2).

**Table 2:** Distribution of participants' knowledge of the role of physiotherapists in various aspects of obstetrics and gynaecology.

No	Variable	Frequency (percentage)				
		Strongly agree	Agree	Neutral	Disagree	Strongly disagree
1.	Urinary incontinence	18 (28.6)	16 (25.4)	22 (35.0)	7 (11.0)	0 (0.0)
2.	Dyspareunia	8 (12.9)	32 (51.6)	12 (19.4)	8 (12.9)	2 (3.2)
3.	Anal incontinence	11 (17.5)	39 (61.9)	9 (14.2)	2 (3.2)	2 (3.2)
4.	Sexual dysfunction	11 (17.4)	37 (58.7)	12 (19.1)	1 (1.6)	2 (3.2)
5.	Urinary tract infection	0 (0.0)	3 (4.8)	25 (39.7)	27 (42.8)	8 (12.7)
6.	Uterine prolapse	17 (27.0)	24 (38.1)	18 (28.5)	2 (3.2)	2 (3.2)
7.	Vaginal prolapse	8 (12.7)	45 (71.4)	4 (6.4)	5 (7.9)	1 (1.6)
8.	Obstetric fistula	5 (7.9)	17 (27.0)	22 (35.0)	14 (22.2)	5 (7.9)
9.	Pelvic inflammatory disease	0 (0.0)	1 (1.7)	23 (39.7)	27 (46.6)	7 (12.0)
10.	Hysterectomy	0 (0.0)	12 (19.0)	26 (41.3)	17 (27.0)	8 (12.7)
11.	Cervical incompetence	0 (0.0)	9 (14.3)	23 (36.5)	21 (33.3)	10 (15.9)
12.	Muscle weakness	42 (67.8)	18 (29.0)	2 (3.2)	0 (0.0)	0 (0.0)

Clinicians expressed varied perceptions of the role of physiotherapy in the care of obstetric and gynaecological patients (Table 3). It was noted that 66.7% disagreed while 27.0% strongly disagreed that physiotherapy may not contribute significantly to the complete wellbeing of obstetric patients. Among the study participants, 50.8% disagreed while 14.3% strongly disagreed that physiotherapy is too expensive to be afforded by their patients. A majority (50.8%) of participants agreed that physiotherapy is time demanding, and 36.5% agreed that physiotherapists should be allowed to attend to patients in the labour ward.

**Table 3:** Participants' attitude about physiotherapy service in obstetric and gynaecological patients.

No	Variable	Frequency (percentage)				
		Strongly agree	Agree	Neutral	Disagree	Strongly disagree
1.	Physiotherapy may not contribute significantly to the complete well‐being of an Obstetric patient	0 (0.0)	3 (4.7)	1 (1.6)	42 (66.7)	17 (27.0)
2.	Physiotherapy is too expensive to be afforded by my patients	0 (0.0)	9 (14.3)	13 (20.6)	32 (50.8)	9 (14.3)
3.	Physiotherapy is time‐demanding.	0 (0.0)	32 (50.8)	6 (9.5)	17 (27.0)	8 (12.7)
4.	Physiotherapists should be allowed to attend the labour ward.	2 (3.2)	23 (36.5)	17 (27.0)	17 (27.0)	4 (6.3)
5.	Physiotherapists should be allowed to attend some surgical operations for gynaecological patients.	0 (0.0)	14 (22.2)	24 (38.1)	16 (25.4)	9 (14.3)
6.	Physiotherapists are not competent to manage my patients,	0 (0.0)	7 (11.3)	17 (27.4)	38 (61.3)	0 (0.0)
7.	Physiotherapy will cause harm to my patients	0 (0.0)	0 (0.0)	10 (15.9)	28 (44.4)	25 (39.7)
8.	Physiotherapists have been adequate in their interprofessional relationships	4 (6.4)	31 (49.2)	14 (22.2)	14 (22.2)	0 (0.0)
9.	Physiotherapy will not benefit my patients	0 (0.0)	3 (4.8)	8 (12.7)	35 (55.5)	17 (27.0)
10.	Physiotherapy will worsen my patients' condition	1 (1.6)	0 (0.0)	0 (0.0)	37 (58.7)	25 (39.7)

**Figure 2:**
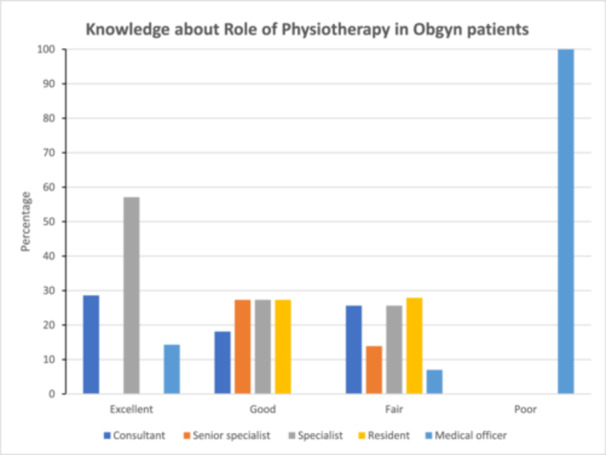
Knowledge about the role of physiotherapy among various ranks of participants.

Findings from the study indicated that consultants (28.6%–excellent), specialists (57.1%–excellent), and senior specialists (27.3%–good) were more knowledgeable on the role of physiotherapy in obstetrics and gynecology than residents and medical officers, as illustrated in the Figure 2.

The study found that 71.4% of clinicians reported having referred patients for physiotherapy. Such referrals were on a weekly (4.8%), monthly (31%), or yearly (34.9%) basis (Table 4). Among the respondents, 54.0% were referred with prescription only. The study established that factors that may influence a clinician's decision to refer patients for physiotherapy interventions include prior knowledge of it during training (50.0%), and previous experience with physiotherapists in managing obstetric (29.5%) and gynaecological (34.4%) patients (Table 4).

**Table 4:** Participants' practice of physiotherapy services in obstetrics and gynaecology.

Practices of participants		Frequency	Percentage (%)
Regularity of patient referral	Daily	0	0.0
	Weekly	3	4.7
	Monthly	20	31.8
	Yearly	22	34.9
	Never	18	28.6
Mode of referral	With prescription only	27	54.0
	Verbal only	0	0.0
	Prescription and verbal	20	40.0
	None	3	6.0
Perception of physiotherapy management of obs/gyn patients	Excellent	3	4.8
	Fair Very Good	8	12.7
	Good	21	33.3
	Very good Fair	29	46.0
	Poor	2	3.2
Factors that can influence referral for physiotherapy			
Cost of physiotherapy	Yes	12	19.1
	No	29	46.0
	Not sure	22	34.9
Prior knowledge of physiotherapy during training	Yes	30	50.0
	No	16	26.7
	Not sure	14	23.3
Presence of physiotherapy unit within the vicinity of discharged clients	Yes	48	80.0
	No	12	20.0
	Not sure	0	0.0
Have worked with physiotherapists in mgt of gynaecological patients before	Yes	21	34.4
	No	22	36.1
	Not sure	18	29.5
Have worked with physiotherapists in mgt of obstetric patients before	Yes	18	29.5
	No	28	45.9
	Not sure	15	24.6

## 4 Discussion

The study revealed that the majority of study participants were males (82%), reflecting male dominance in this clinical area. This is consistent with the findings of Odunaiya et al. [9]. The majority of the clinicians have had very high and specialized training in this field of practice, which is typical of a teaching hospital of this caliber in Ghana.

The study established that consultants, specialists, and senior specialists were more knowledgeable about the role of physiotherapy in obstetrics and gynecology than residents. These findings agree with what was reported by Odunaiya et al. Years of experience in the profession is likely to account for this disparity among clinicians. The majority of participants knew of the role of physiotherapists in obstetrics and gynaecology in managing conditions such as muscle weakness, vaginal prolapse, anal incontinence, and sexual dysfunction. However, the low score observed for knowledge of the role of physiotherapy in pelvic inflammatory disease and hysterectomy indicates that although obstetricians and gynecologists had a general knowledge of the role of physiotherapy service, they had limited knowledge regarding specific conditions, as corroborated by Odunaiya et al. [9]. This also shows that obstetricians and gynaecologists have not been involving physiotherapists in the management of conditions such as pelvic inflammatory disease.

Most clinicians agreed that patients with obstetric and gynecological conditions also require physiotherapy services, and that physiotherapy would not cause harm to them. Although about half of the clinicians admitted physiotherapy could be time‐consuming, it was inexpensive and can be easily afforded by patients. Study participants varied in opinions, with a little over a third indicating that physiotherapists should be allowed to attend to cases in the labour ward. It is worth noting that numerous factors could account for this finding. The labour ward setting may not be well‐resourced to provide optimum privacy for patients, and hence, such services at the time of labour may be considered by some clinicians as interference. Others may believe that this should be possible without causing any interference. This is in agreement with other studies [7, 9]. Under‐utilization of physiotherapy may be due to limited knowledge of the part of obstetricians and gynecologists about the role of physiotherapists in parturition. Further, 22.2% of participants disagreed that physiotherapists have performed adequately in their interprofessional relationships. This is indicative of a need for improved communication in the form of seminars, workshops, and attendance at grand rounds.

Almost 8 out of every 10 participants referred patients for physiotherapy. This practice could be due to the level of knowledge and attitude, and hence improved utilization of physiotherapy services. A greater proportion of clinicians who refer patients for physiotherapy indicated they do so because of the presence of physiotherapy units within the vicinity of discharged clients. This may be due to a nationwide increase in awareness of physiotherapy interventions and services, hence an increase in the establishment of physiotherapy units.

## 5 Conclusion

Obstetricians and gynaecologists at Komfo Anokye Teaching Hospital had a general knowledge about the role of physiotherapy in obstetrics and gynaecological practice but lacked knowledge of specific conditions such as pelvic inflammatory disease, and hysterectomy. Clinicians have not adequately utilized the services of physiotherapists in taking care of their obstetric and gynaecological patients.

Regular interactions between obstetricians, gynecologists, and physiotherapists to enhance a multi‐disciplinary approach to health care delivery in such settings are highly recommended. This can be enhanced through seminars, workshops, and grand rounds. Researchers can also consider undertaking further research based on the hypotheses generated to measure any correlations or associations.

### 5.1 Limitations of Study

The study reported on a small sample size. Again, most of the variables (knowledge, practice) that were assessed were self‐reported by respondents and not empirically measured. The adapted questionnaire has not been used or validated in the settings before this study. This is also a limitation. In spite of these limitations, rigorous data collection and analysis was employed to generate these findings. The findings of the study can be applied to settings like the current study site.

## Author Contributions

**Seth Amponsah‐Tabi:** conceptualization, investigation, supervision, data curation, writing – review and editing, project administration, and methodology. **Daniel Awli Mawunyo:** conceptualization, methodology, and writing – original draft. **Timothy Kwabena Adjei:** writing – review and editing, data curation, and visualization. **Cosmos Yarfi:** data curation, software, formal analysis, methodology, and conceptualization. **Amponsah Peprah:** formal analysis and investigation. **Maxwell Kankam:** validation and visualization. **Charles Senaya:** methodology, investigation, and supervision. **Jude John Annan:** supervision, data curation, formal analysis, project administration, writing – review and editing. **Roderick Larsen Reindorff:** supervision, project administration, writing – review and editing.

## Conflicts of Interest

The authors declare no conflicts of interest.

## Transparency Statement

The lead author Seth Amponsah‐Tabi affirms that this manuscript is an honest, accurate, and transparent account of the study being reported; that no important aspects of the study have been omitted; and that any discrepancies from the study as planned (and, if relevant, registered) have been explained.

## Data Availability

All data generated or analyzed during this study are included in this article and its supplementary information data can be requested from corresponding author.
